# CBCT-Based Anthropometric Evaluation of Edentulous Alveolar Crest Lengths

**DOI:** 10.3390/diagnostics15121525

**Published:** 2025-06-16

**Authors:** Ozgun Yildirim, Eda Izgi, Mustafa Ozturk, Orhan Gulen

**Affiliations:** 1 Department of Oral and Maxillofacial Surgery, Gülhane School of Dentistry, Health Sciences University, 06010 Ankara, Turkey; 2 Department of Oral and Maxillofacial Surgery, School of Dentistry, Kütahya Health Sciences University, 43020 Kütahya, Turkey; eda.izgi@ksbu.edu.tr; 3 Private Dental Clinic, 06510 Ankara, Turkey; mstozturk44@hotmail.com; 4 DentisTomo Dental Imaging Center, 06420 Ankara, Turkey; orhangulen@gmail.com

**Keywords:** age, alveolar process, augmentation, block onlay allografts, cone beam computed tomography, gender

## Abstract

**Objectives**: With technological advancements, fabricated block onlay allografts have been developed, particularly for grafting severely resorbed alveolar crests before implant surgery. This study aimed to evaluate the alveolar crest lengths of the edentulous maxilla and mandible using cone beam computed tomography (CBCT) and to investigate their correlations with gender and age. The data obtained will serve as preliminary input for block onlay allograft production. **Methods**: CBCT scans of 451 participants and 595 edentulous jaws were analyzed. The following measurements were taken: the distance between the zygomatic buttress projection and the infraorbital foramen projection onto the crest (U1, U4); from the infraorbital foramen projection to the incisive canal (U2, U3); between the midpoint of the linea obliqua externa projection and the mental foramen projection (L1, L4); and from the mental foramen projection to the midline at the incisive canal level (L2, L3). Measurements were compared across gender and age groups. **Results**: No significant differences were observed in maxillary measurements between genders. However, the values of L1, L2, and L4 in males were significantly higher than those in females (*p* < 0.05). Age did not affect substantially most measurements, except for L4, where individuals over 70 years had lower mean values than those under 50 years (*p* < 0.05). **Conclusions**: The mandibular posterior region demonstrated the most prominent residual alveolar crest. Mandibular crest lengths were significantly greater in males. Although age showed a limited impact, a reduction in posterior mandibular crest length was evident in individuals over 70 years compared to younger individuals.

## 1. Introduction

Following tooth extraction, the alveolar crest undergoes progressive and often irreversible resorptive changes that significantly alter its morphology. This remodeling process reduces the volume and continuity of the residual alveolar ridge, posing a challenge for prosthetic and implant-based rehabilitation [1]. Understanding the morphological characteristics of edentulous alveolar crests—particularly their linear dimensions—is essential for clinical planning, especially in cases requiring ridge augmentation or implant placement [2].

Optimal functional and aesthetic prosthetic rehabilitation after successful implant treatment is only possible when sufficient residual alveolar bone volume is available. However, following tooth loss, a series of resorptive changes occur that result in a loss of alveolar crest volume [3]. Tooth loss causes atrophy of the alveolar crest, a chronic, progressive, irreversible, and cumulative process [4]. According to Frost’s mechanostat theory on the subject, long-term tooth loss leads to a lack of mechanical stimulation due to the absence of tooth use, resulting in a reduction in the volume of the residual alveolar crest [5].

Morphological variations in the maxilla and mandible are often influenced by age, gender, ethnicity, environmental factors, degree of atrophy, and differences in assessment techniques [6,7,8]. Dental factors, such as periodontitis, periapical pathologies, and traumatic tooth extractions, can also lead to alveolar bone damage [1,2,9]. Changes in bone form and quantity in the alveolar crest affect the availability of bone for implant treatment in edentulous patients. Therefore, a detailed clinical and radiological examination is essential in placing dental implants in the appropriate size and ideal position and reducing the risk of damage to neighboring anatomical structures [6]. CBCT images allow a precise examination of the position of anatomical structures and provide clinicians with detailed information about bone morphology and sinus pathologies, which are crucial for accurate dental implant planning leading to success.

Nowadays, depending on the development of technology, personalized block onlay allografts are produced; however, there is also a need for fabricated block onlay allografts compatible with alveolar crest shapes. There is a need for scientific data on the arch length that blocks onlay allografts to shorten the surgical procedure’s duration and increase graft operations’ success. This study aimed to determine the bone lengths of edentulous residual alveolar crests in the maxilla and mandible using cone beam computed tomography (CBCT) scans and to investigate the correlations between the obtained data and different gender and age groups in the Turkish population.

Although previous studies have explored ridge morphology using CBCT, most have focused on the vertical height or cross-sectional anatomy, with a limited emphasis on linear alveolar crest lengths spanning anatomical landmarks across the edentulous arch. This study addresses this gap by providing population-specific quantitative data on alveolar crest lengths in the maxilla and mandible, which may serve as a baseline for future anthropometric research and inform the design parameters of prefabricated graft materials.

## 2. Methods

### 2.1. Study Population

For this retrospective observational study, the population was obtained from a convenience sample randomly selected from patients referred to a radiology center (DentisTomo Dental Imaging Centre, Ankara, Türkiye) for CBCT with various indications between July 2024 and September 2024. Radiographs were included in the analysis according to the following criteria for participants: (1) patients older than 20 years; (2) completely edentulous maxilla and mandible; (3) CBCTs obtained from individuals in the Turkish population; (4) radiographs without any pathological findings; (5) all CBCT images were obtained with the same device and at the same time; and (6) at least two years had passed since tooth extractions and other surgical procedures were performed. Radiographs were excluded from the analysis if at least one of the following criteria was positive: (1) CBCTs in which only one jaw was imaged; (2) the presence of teeth in the jaws, even a single tooth; (3) the presence of radiolucent or radiopaque intraosseous lesions associated with pathological conditions; (4) partial or total bone retention of 1 or more residual roots; (5) the presence of dental implants; (6) radiographic evidence of bone augmentation procedures or signs of invasive surgery; (7) the presence of osteosynthesis plates; and (8) unreadable CBCTs.

Out of 856 archived CBCT scans screened retrospectively, 451 radiographs met all inclusion criteria and were included in the study. The relatively short time frame was feasible due to the high daily imaging volume at the radiology center and the retrospective nature of data retrieval.

Within the pool of eligible CBCT scans that met all inclusion criteria, simple random selection was performed using a computer-generated random number list to ensure unbiased inclusion from the convenience sample.

One researcher with 20 years of experience as a maxillofacial radiologist analyzed all images. To avoid getting tired, the researcher worked five days a week, between 13.00 and 15.00. After two weeks, 20% of the evaluations was re-evaluated.

### 2.2. Processing of CBCT Scans and Radiographic Measurements

**Figure 1:**
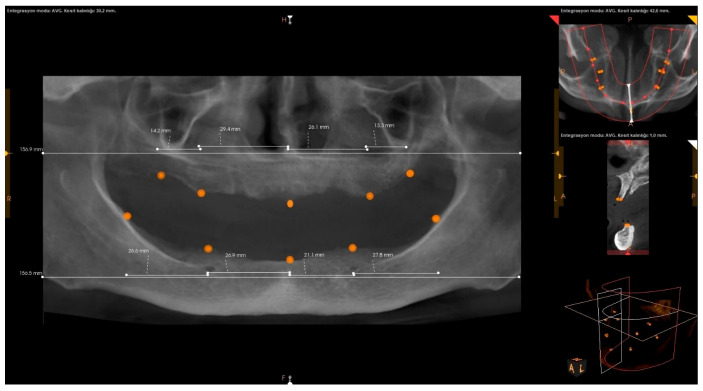
Landmarks on a panoramic radiograph.

**Figure 2:**
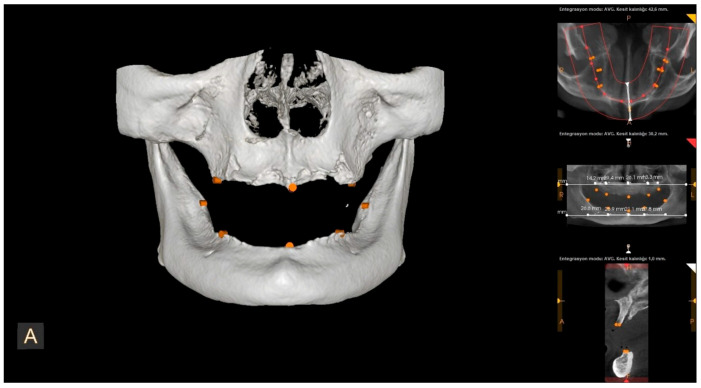
Landmarks on a CBCT image (front view).

**Figure 3:**
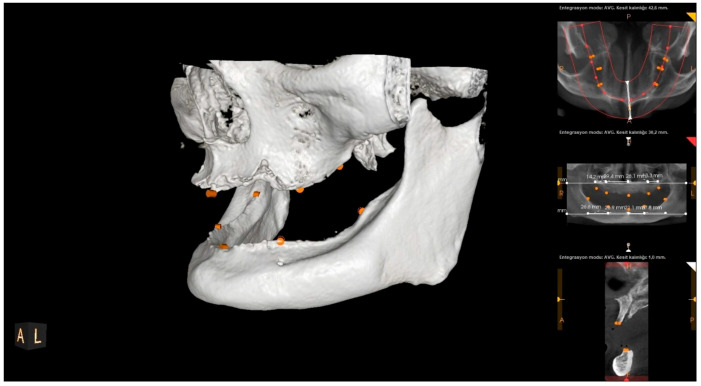
Landmarks on a CBCT image (left side view).

**Figure 4:**
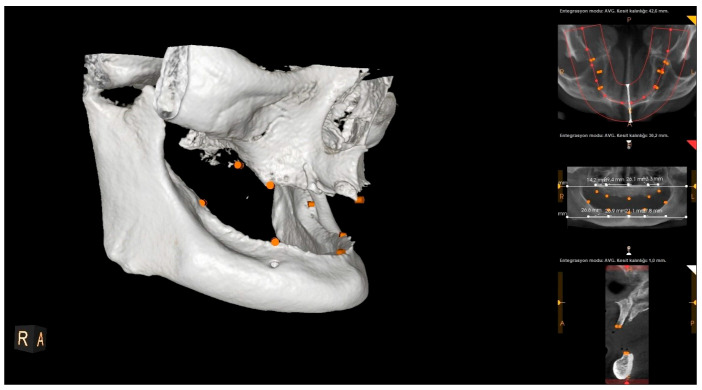
Landmarks on a CBCT image (right side view).

All CBCT scans were obtained using an HDX brand Dentri-Sα (Seul, South Korea) device. The irradiation parameters were 80–90 kVp and 7–10 mA, and the imaging area was 16 × 14.5–16 × 8 mm (free FOV). Specific anatomical landmarks were used for the measurement areas in the maxilla and mandible (Figure 1). These measurement areas are as follows: the distance between the projection of the right zygomatic buttress to the crest and the projection of the right infraorbital foramen to the crest (U1); the distance from the projection of the right infraorbital foramen to the crest to the mouth of the incisive canal (U2); the distance between the incisive canal and the projection of the left infraorbital foramen (U3); the distance between the projection of the left infraorbital foramen and the projection of the left zygomatic buttress (U4); the distance between the midpoint of the projection of the right linea oblique externa and ramus and the projection of the right foramen mentale onto the crest (L1); the distance between the projection of the right foramen mentale and the midline point at the level of the incisive canal (L2); the distance between the midline point at the level of the incisive canal and the projection of the left foramen mentale (L3); and the distance between the projection of the left foramen mentale and the midpoint of the projection of the left linea oblique externa (L4) (Figure 2, Figure 3 and Figure 4). The data obtained after the measurements were transferred to the computer (Microsoft Excel, 2013), and the relationship between the measurements and the age and gender groups were evaluated separately.

### 2.3. Statistical Analysis

#### 2.3.1. Data Analysis

IBM The Statistical Package for Social Sciences (SPSS) Statistics version 28 Windows package was used to analyze the data in the study. Descriptive statistical methods (numbers, percentages, minima, maxima, and medians) were used to evaluate the data. In this study, whether there was a statistically significant difference between mandibular and maxillary arch length measurements according to gender was tested by the independent sample t-test, and the statistical relationships between age groups were tested by Pearson’s correlation analysis. All analyses were performed with a 95% confidence interval, and the Type I error probability was set to α = 0.05.

#### 2.3.2. Sample Size

The first hypothesis of this study was analyzed by the independent sample t-test. The sample size required to evaluate the hypothesis was calculated using the “GPower-3.1.9.2” program. The sample size was calculated using Cohen’s standardized effect size with a 95% confidence level, α = 0.05, and 0.95 theoretical power since there were no similar studies in the literature [10]. Medium effect sizes were calculated. As a result of the analysis performed with the Gpower program, the minimum sample size was calculated as 105 for each group and 210 in total.

This study’s second hypothesis regarding whether there are statistically significant relationships between age and the measurements was tested using a correlation analysis. The required sample size for the hypothesis was calculated using the “GPower-3.1.9.2” software. The sample size was determined based on a 95% confidence level, α = 0.05, and 0.95 theoretical power using Cohen’s medium effect size, as there were no similar studies in the literature to standardize the effect size. The analysis conducted with the G*Power software (GPower-3.1.9.2) determined that the minimum sample size required was 138.

When the hypotheses were analyzed, it was decided that a sample number of 210 was appropriate to investigate both hypotheses.

#### 2.3.3. Intraobserver Repeatability

Statistical Method: The SPSS Windows version 25 (SPSS Inc. Chicago, IL, USA) software program was used to evaluate the data in this study. The Intraclass Correlation Coefficient (ICC) was used to assess the consistency between the lower and upper jaw measurements. The ICC provides information about the consistency and reliability of the measurements. The data were analyzed in a design where each measurement was repeated on the same individual. ICC values vary between 0 and 1, and the consistency between the measurements increases as the value increases. As a result of the analysis, *p*-values were calculated along with the ICC values, and *p*-values less than 0.05 indicated that the consistency was statistically significant. The Type I error probability was determined as α = 0.05 for all analyses. The ICC was used to evaluate the intraobserver consistency of repeated measurements for each anatomical region (maxilla and mandible separately), not to compare the two areas.

ICC values for the lower jaw ranged from 0.65 to 0.85. Accordingly, high consistency was observed for L1 (ICC = 0.849) and L4 (ICC = 0.724), while moderate consistency was observed for L2 (ICC = 0.695) and L3 (ICC = 0.650). All ICC results were statistically significant (*p* < 0.05).

ICC values for the upper jaw ranged from 0.45 to 0.88. Accordingly, high consistency was observed for U2 (ICC = 0.884), moderate consistency for U1 (ICC = 0.618) and U3 (ICC = 0.676), and lower consistency for U4 (ICC = 0.449). All ICC results were statistically significant (*p* < 0.05).

### 2.4. Compliance with Reporting Guidelines

This retrospective cross-sectional study was conducted and reported according to the STROBE (Strengthening the Reporting of Observational Studies in Epidemiology) guidelines.

### 2.5. Ethical Approval

The Kütahya Health Sciences University Non-Interventional Clinical Research Ethics Committee reviewed and approved this study (Decision No: 2024/09-04, 16 July 2024).

## 3. Results

### 3.1. Study Population

When the distribution of the participants according to gender was analyzed, it was found that 57.4% were female and 42.6% were male. When the distribution of the participants according to age was analyzed, it was found that 13.3% were under 50 years, 32.4% were 51–59 years, 39.5% were between the ages of 60 and 69 years, and 14.9% were 70 years and older. The mean age of the participants was 60.05 ± 9.76 years. It was determined that 33.5% of the participants’ CBCT extraction sites were the mandible, 33% were the maxilla, and 33.5% were both the maxilla and mandible.

The Mann–Whitney U test was applied to assess whether there was a statistically significant difference in participant age between the male and female groups, as the age data did not follow a normal distribution. According to the results of the analysis, it was determined that there was a statistically significant difference between the ages according to gender (*p* < 0.05). Accordingly, it was observed that the median value of the age of women was higher than that of men (Table 1).

Of the 451 participants included in the study, 156 were edentulous in both jaws, while others were edentulous in only the maxilla or mandible. As a result, measurements were conducted on 296 maxillae and 299 mandibles. The differences in ‘*n*’ values reflect the availability of edentulous jaw segments suitable for measurement using CBCT.

### 3.2. Radiographic Measurements

Descriptive statistics of the measurement values in the maxilla and mandible are presented in Table 2. The distribution of measurement values according to gender and age groups is shown in Table 3 and Table 4.

Whether there were statistically significant differences in the values of the measurement areas determined in the maxilla and mandible according to gender was tested by the independent sample *t*-test for normally distributed data and the Mann–Whitney U test for non-normally distributed data (Table 3). As a result of the analysis, it was determined that there were no statistically significant differences in the U1, U2, U3, U4, and L3 values between male and female participants (*p* > 0.05), while there were significant differences in the L1, L2, and L4 values between genders (*p* < 0.05), with higher values recorded in males. Accordingly, it was observed that the mean values of L1, L2, and L4 in women were lower than those in men.

Whether there were statistically significant differences in the values of the measurement areas determined in the maxilla and mandible according to the age groups was tested by analysis of variance (ANOVA) for normally distributed data and the Kruskal–Wallis test for non-normally distributed data (Table 4). As a result of the analysis, no statistically significant differences between age groups were found for the U1, U2, U3, U4, L1, L2, and L3 values (*p* > 0.05). However, a significant difference was observed in the L4 measurements (*p* < 0.05). The post hoc Bonferroni analysis revealed that participants aged 70 years and older had significantly lower L4 values than those aged 50 years and younger.

## 4. Discussion

Dental implant operations are one of the most preferred methods in rehabilitating missing teeth to provide aesthetics, function, and phonation. The biomechanically appropriate positioning of the dental implant depends on the amount and quality of bone available in the placement area [11]. While bone grafting decisions are inherently patient-specific—depending on factors such as the duration of edentulism, history of denture use, timing of extractions, and prior pathologies—the descriptive morphometric data presented in this study may serve as a helpful reference point during initial treatment planning, particularly in cases where rapid prototyping or prefabricated graft options are considered. These measurements do not replace individualized planning but may inform baseline expectations and dimensional ranges in populations with similar demographic characteristics.

Recently, multi-slice and high-resolution images can be obtained with CBCT [12]. Its use in volumetric measurements has been defined as the gold standard. Its accuracy has been proven with a relative error margin of less than 1% compared to measurements made with digital calipers [4]. In clinical practice, panoramic radiographs are the first and most commonly used method for imaging before dental implant surgery. Panoramic radiography is used to evaluate the general shape of the jaw and the basic anatomical structures (foramen mentale and mandibular canal). Anatomical landmarks and existing teeth can be reference points in panoramic radiographs. However, the disadvantages of panoramic radiographs are the lack of three-dimensional images, low resolution, high distortion, and the presence of phantom images. In addition, the mean linear error of panoramic radiographs in a volumetric examination of bone was 24%, and the mean linear error of CBCT was 1.8% [8]. Peker et al. concluded that measurements obtained from CBCT images are more consistent with direct measurements than measurements obtained from panoramic radiographic or conventional tomographic images [13]. Therefore, in this study, CBCT images were utilized for the volumetric examination of edentulous alveolar crests. In future studies, a three-dimensional volumetric analysis and cross-sectional assessments of the alveolar crest will be performed using advanced segmentation software. This will allow a more complete characterization of the alveolar morphology, which is crucial for precision-engineered graft fabrication.

Residual crest resorption reaches a maximum within 2 years after tooth extraction and decreases gradually thereafter [14]. The change in crest height and width averages 2.03 mm and 3.87 mm, respectively, after a period of 3 to 12 months after tooth extraction [15]. It is conceivable that the ridge dimensions reported here were obtained from a group of edentulous patients in whom the significant dimensional changes in the ridge occurred 2 years after tooth loss. Understanding the physiological process of bone remodeling after tooth extraction and a comprehensive three-dimensional evaluation of the recipient site before implant placement is recommended for optimal implant planning and to minimize the risk of possible complications [16].

In cases where the residual alveolar bone volume is insufficient for implant placement, regenerative or reconstructive methods provide the ideal bone volume and anatomical conditions [17]. Although satisfactory results have been reported with short, angled, zygomatic, and pterygoid implants in areas with an insufficient bone volume, reconstruction remains the best treatment option for these areas [18]. Reconstructive techniques for placing dental implants include maxillary sinus augmentation, guided bone regeneration, osteogenic distraction, split-crest grafts, and onlay/inlay bone grafts [17]. Since planning on CBCT will be one of the most critical points leading to success when deciding on the reconstruction technique, this study aimed to evaluate the alveolar crest volume of the edentulous maxilla and mandible in the Turkish population. In light of the data obtained from this study, the planning of fabricated block onlay allograft production will be a preliminary preparation for the following research. If 5 mm width and 10 mm height are considered ideal for implant placement in dental arches, fabricated onlay allografts can be produced with the arch length data obtained from this study. Different analyses can be performed in subsequent studies.

It is the gold standard in autologous bone reconstruction due to its osteogenic, osteoinductive, and osteoconductive properties. However, a second surgical site brings disadvantages such as the risk of complications, morbidity, and difficulty of use [19]. To overcome these disadvantages, many clinicians utilize allografts and xenografts. Allografts and xenografts are widely used because they can be obtained in the desired amount and guide the growth and proliferation of osteoblasts on their surface. However, the disadvantages of these grafts are low resorbability and the potential for infectious diseases [20].

With the introduction of computer-aided design/computer-aided manufacturing (CAD/CAM) techniques in dentistry, developments that contribute to bone regeneration have accelerated [21,22]. The determination of the residual bone volumes of patients using CBCT has made it possible to obtain 3D models of the jaws. It has become possible to reconstruct the missing bone areas on these models and to design and produce patient-specific onlay grafts by a milling technique or 3D printing [17,22]. To take this development further, in this study, the distribution of residual crest widths in the maxilla and mandible in different age and gender groups was evaluated in a cross-section taken from the Turkish population. In light of the data obtained in this way, the production of fabricated onlay allografts with a pilot study planned in the future will shorten the time taken for custom onlay graft production, reduce the cost, and increase the working comfort of the clinician.

Analyzing residual alveolar crest values for different dentition states, genders, and age groups is crucial in clinical practice. These data provide a critical basis for assessing the risk of bone volume loss and developing appropriate treatment strategies accordingly. Anatomical differences between the sexes [9,23], age effects [23,24], and ethnic differences [25] have been noted. It has been determined that factors such as the alveolar crest components contribute to statistical heterogeneity by affecting other alveolar crest components. In the analysis of the results in this study, no statistically significant difference was found between the age groups in the measurements of the maxilla and mandible. However, a significant difference was found in the right posterior mandible region. To determine in which age groups this difference occurred, it was identified that the average value for participants aged over 70 years was lower compared to participants aged 50 years and younger. These differences may be attributed to variations in this study’s methodology, sample size, and age range. In line with our findings, another study that conducted a radiomorphometric analysis of the edentulous posterior mandible in the Turkish population reported negative correlations between age and crest width, height, and lingual concavity depth [26]. In other studies, no significant differences were found between age groups in the alveolar crest measurements [6,26,27].

In a study by Couso-Queiruga et al. evaluating the dimensions of the alveolar crest after extraction, it was concluded that after a healing period of 2 to 9 months, there was a greater dimensional reduction in all directions in molar regions compared to the other areas [28]. Alveolar crest resorption is more pronounced in the horizontal dimension, followed by vertical changes. Non-molar sites require more hard tissue augmentation methods than molar sites before or during implant placement. No significant difference was found between genders in radiographic measurements of edentulous alveolar crests. In this study, the results obtained for the length measurements of edentulous alveolar crests in the maxilla did not differ between males and females. In the mandible, the results obtained for the width measurement of the posterior residual crest showed that males had a statistically significantly longer crest than females. This finding suggests that gender may have a limited effect on the process of alveolar crest resorption. Studies on the symmetry of the dental arches have shown that the right and left dental arches are similar in size or shape in different gender groups, with no statistically significant differences [29,30]. This study found no statistically significant differences between the right and left segments for the measurements made in the maxilla and mandible. Given the significant gender-related differences in mandibular crest lengths, particularly in the posterior region (L1, L2, and L4), future allograft designs may benefit from adjusting block dimensions based on the patient’s sex. For example, blocks for male patients may require slightly longer dimensions in the posterior mandible to ensure complete coverage and stability.

This study provides CBCT-based anthropometric data on the residual alveolar crest lengths in edentulous maxillae and mandibles in a Turkish population, with specific reference to gender- and age-related variability.

Another limitation is the absence of interobserver reliability analysis. All measurements were performed by a single experienced radiologist, which may introduce operator bias despite the high intraobserver agreement for most variables. Also, information on participants’ systemic health, bone metabolism, or comorbidities (e.g., osteoporosis and diabetes) was unavailable, which may confound interpretations of anatomical variability.

Finally, intraobserver agreement was generally acceptable. The lower ICC value for U4 indicates the need for more robust and standardized measurement techniques. To improve reliability, future studies will include multiple raters and enhanced imaging protocols.

## 5. Conclusions

This CBCT-based anthropometric study provides baseline data on alveolar crest lengths in edentulous maxillae and mandibles in a Turkish population, considering gender- and age-related differences. The findings may serve as a foundational reference for future population-specific or ethnic studies and could potentially inform subsequent research on graft planning or prosthetic rehabilitation strategies. However, the results are not intended for direct clinical application without an individual assessment. Future studies should include larger, multicenter, prospective observational investigations to validate these anatomical measurements across different populations and ethnic groups. Once standardized morphometric data are established, subsequent interventional studies may be warranted to evaluate the clinical effectiveness of fabricated allografts designed based on these data.

The crest dimensions reported in this study were obtained from a group of edentulous patients in whom the crest’s major volume and shape changes occurred after tooth loss. Fabricated block onlay allografts of specific length scales are planned for a future study. Therefore, not including alveolar crest height measurements in this study can be considered one of its limitations. Furthermore, future longitudinal studies are warranted to better distinguish between age-related changes and variations in alveolar crest dimensions associated with post-extraction remodeling.

In the next phase of our research, we plan to fabricate block onlay allografts based on the dimensional data from this study and test their clinical efficacy in a randomized controlled trial. This trial will assess graft stability, surgical efficiency, and bone integration outcomes in edentulous patients.

## Figures and Tables

**Table 1:** Comparison of the ages of the participants according to gender.

	Gender	*n*	Median (Min.–Max.)	Center ± S.s.	Test Statistic	*p*
Age	Female	259	61 (34–86)	61.05 ± 9.79	U = 21,489.500	0.014 *
	Male	192	59 (22–87)	58.7 ± 9.57		

*U: Mann–Whitney U test statistic*, * *p < 0.05*.

**Table 2:** Descriptive statistics of measurement area variables.

Measurement Fields	*n*	Median (Min.–Max.)	Center ± S.s.
U1	296	13 (6.6–29.6)	13.47 ± 3.15
U2	296	24.85 (11.6–36.5)	25.02 ± 4.04
U3	296	24.25 (9.6–35.8)	24.41 ± 3.93
U4	296	12.7 (5.7–29.3)	13.05 ± 3.06
L1	299	30.6 (16–45.1)	30.74 ± 4.59
L2	299	23.6 (12.2–33.3)	23.2 ± 3.44
L3	299	25.2 (9.5–36.8)	24.97 ± 3.46
L4	299	28.7 (16–41.6)	28.49 ± 4.13

**Table 3:** Comparison of measurement areas according to gender.

Measurement Fields	Gender	*n*	Median (Min.–Max.)	Centrer ± S.s.	Test Statistic	*p*
U1	Female	165	12.9 (7–29.6)	13.25 ± 2.89	U = 10,201.500	0.407
	Male	131	13.3 (6.6–25.4)	13.73 ± 3.43		
U2	Female	165	24.9 (12.7–36.5)	25.2 ± 3.88	t = 0.877	0.381
	Male	131	24.5 (11.6–35.8)	24.79 ± 4.24		
U3	Female	165	24.2 (9.6–35.8)	24.34 ± 3.87	U = 10,479.000	0.653
	Male	131	24.3 (10.7–35.2)	24.49 ± 4.03		
U4	Female	165	12.7 (7.9–23.1)	13.1 ± 2.88	U = 10,278.500	0.469
	Male	131	12.7 (5.7–29.3)	12.99 ± 3.28		
L1	Female	176	30.05 (16–42.2)	29.79 ± 4.3	t = −4.386	0.000 *
	Male	123	31.5 (22–45.1)	32.09 ± 4.66		
L2	Female	176	23.25 (12.2–33.1)	22.85 ± 3.27	t = −2.156	0.032 *
	Male	123	24.1 (14.6–33.3)	23.71 ± 3.63		
L3	Female	176	24.65 (9.5–36.2)	24.67 ± 3.61	t = −1.835	0.068
	Male	123	25.6 (17.6–36.8)	25.41 ± 3.21		
L4	Female	176	27.45 (16–41.6)	27.58 ± 4.11	t = −4.698	0.000 *
	Male	123	30.2 (19.8–39.7)	29.78 ± 3.81		

t: Independent sample t-test, U: Mann–Whitney U test statistic, * p < 0.05.

**Table 4:** Comparison of measurement areas according to age groups.

Measurement Fields	Age	*n*	Median (Min.-Max.)	Center ± S.s.	Test Statistic	*p*	Bonferroni (*p*)
U1	Under 50 years old	45	12.9 (6.6–29.6)	13.13 ± 3.43	KW = 1.434	0.698	-
	50–59 years old	105	13.4 (7.9–25.4)	13.50 ± 2.83			
	60–69 years old	110	12.8 (7–25.3)	13.48 ± 3.27			
	70 years old and above	36	13.25 (7.6–22.3)	13.75 ± 3.35			
U2	Under 50 years old	45	25.4 (18.4–35.8)	25.35 ± 3.77	F = 1.868	0.135	-
	50–59 years old	105	24.9 (13.6–35.5)	24.87 ± 3.76			
	60–69 years old	110	24.1 (11.6–34.6)	24.59 ± 4.31			
	70 years old and above	36	26.45 (15.1–36.5)	26.35 ± 4.19			
U3	Under 50 years old	45	24 (17.3–34.3)	24.27 ± 3.66	KW = 1.740	0.628	-
	50–59 years old	105	24.3 (17.4–35.8)	24.63 ± 3.87			
	60–69 years olf	110	23.85 (9.6–35.2)	24.07 ± 4.09			
	70 years old and above	36	24.75 (14.7–35.2)	24.99 ± 4.01			
U4	Under 50 years old	45	12.6 (8.1–17.4)	12.77 ± 2.21	KW = 3.505	0.320	-
	50–59 years old	105	12.8 (7.3–22.9)	13.12 ± 2.75			
	60–69 years old	110	12.6 (7.9–29.3)	12.88 ± 3.49			
	70 years old and above	36	13.55 (5.7–20.9)	13.75 ± 3.40			
L1	Under 50 years old	35	31.1 (21.4–43.5)	31.50 ± 4.93	F = 0.774	0.509	-
	50–59 years old	91	30.5 (22.8–45.1)	30.86 ± 4.14			
	60–69 years old	126	30.6 (17.8–42.2)	30.72 ± 4.70			
	70 years old and above	47	30.3 (16–42)	29.98 ± 4.85			
L2	Under 50 years old	35	23.7 (18.4–29.4)	23.73 ± 2.64	F = 1.433	0.233	-
	50–59 years old	91	23.7 (15.2–32.4)	23.53 ± 3.10			
	60–69 years old	126	23.8 (12.2–33.3)	23.12 ± 3.99			
	70 years old and above	47	22 (16.9–29.6)	22.40 ± 2.90			
L3	Under 50 years old	35	24.7 (18.9–31.9)	24.35 ± 3.06	F = 0.459	0.711	-
	50–59 years old	91	25.4 (16.8–35.7)	25.07 ± 3.32			
	60–69 years old	126	25.25 (9.5–36.2)	25.1 ± 3.62			
	70 years old and above	47	25 (17.7–36.8)	24.9 ± 3.66			
L4	Under 50 years old (1)	35	29.7 (22.5–39.5)	29.45 ± 3.71	F = 2.633	0.050 *	4 < 1 (0.041 *)
	50–59 years old (2)	91	29.10 (18.6–39.7)	28.87 ± 3.65			
	60–69 years old (3)	126	28.45 (16–41.6)	28.45 ± 4.58			
	70 years old and above (4)	47	26.70 (19.6–34.7)	27.13 ± 3.80			

F: Analysis of variance statistic, KW: Kruskal–Wallis test statistic, * Age group 70+ years < age group ≤ 50 years (p = 0.041). The post hoc Bonferroni analysis indicates that participants aged 70 years and older had significantly lower L4 values compared to those aged 50 years and younger.

## Data Availability

All necessary data are provided in the article.
